# 

**Published:** 1855-01

**Authors:** 


### INDEX.

A

Abscesses, M. Jobert on cold 562
Albumen as a cholagogue 572
Albuminuria, cases of, by F. W. Lewis, M.D., 7
Allen, Dr. J. M., The Practical Anatomist, notice 629
Allman, Professor, election of 750
Alison, Dr. W. P., notes on the application of statistics to questions in medical science, &c. 752
American Medical Association, Dr. N. S. Davis's history of, notice 551
American Medical Association's Transactions, notice of 37, 108
Amputation of thigh in civil and military practice 316
Animal and vegetable physiology, by H. C. 198
Animal Decomposition as cause of cholera, by Dr. H. Hartshorne 451
Anthelmintic action of sulphate of quinia 510
Apparatus, new, for fractures of the leg, by J. Neill, M.D. 579
Arsenic, case of poisoning with, by Edward Hartshorne, M.D. 707
Ashwell, Samuel, Treatise on Diseases of Women, notice 419

B

Barnwell, R., on use of the pad in varicose ulcers 504
Barton, Dr. E. H., Cause and Prevention of Yellow Fever, notice 683
Beasley, Mr. Henry, Book of Prescriptions, notice 747
Bell, Dr. John, on mineral and thermal springs of the U. States 333, 394
Bell, Dr. John, Treatise on Mineral and Thermal Springs of the United States, notice 492
Beck, Dr. J. B., Treatise on Infant Therapeutics, notice 232
Betton, Dr. T. F., Address before Philada. Co. Med. Soc., notice 120
Bedford, Dr. G. S., Treatise on Diseases of Women, notice 536
Bigelow, Dr. Jacob, on Nature in Disease, notice 27
Bone, theory of formation of 255

C

Calculus, small one removed 497
Calculous phthisis, M. Forget on 101
Caldwell, Dr. Chas., autobiography of 211
Carter, T. A., on starch corpuscles in body 573
Cataract needle, new form of, by Dr. J. Neill 332
Cataract, operations for, by Geo. Dock, M.D. 583
Camphor as an antidote to strychnia 639
Carpenter, Dr. Wm. B., on the Principles of Human Physiology, &c., notice 744
Cancer, Diagnosis of Surgical, by John Zachariah Laurence, M.D., notice of 745
Card of the committee of prize essays of the American Medical Association 751
Cephalic version in shoulder presentation, by Dr. R. A. F. Penrose 405
Chancre, Prof. Sigmund on 641
Cheeseman, Dr. Andrew, on induction of labor at seventh month 531
Chenol, M. Adrian, on pure oxyde of carbon 186
Chloroformization in midwifery, case of 571
Child-bed Fevers, Treatise on, by C. D. Meigs, notice 18

Cholera morbus, fatal case of, by Dr. J. F. Meigs 402
Cholera 57
Cholera, nature and treatment of, by H. Hartshorne, M.D., 321
Christison, Dr., on preserved meat juice 189
Cholera, Treatise on, by H. G, Jameson, M D., notice 549
Clinical instruction, editorial on 38
Clinical Lectures on Diseases of Women and Children, notice 536
Cochran, Dr. W. S., on obesity in a child 408
Corpuscles, starch, in tissues of the human body 573
Contagion of puerperal fever, by J. C. Morris, M.D. 327
Consumption hospital 750
Correction 315, 442, 554
Coolies, Malabar 58
Costa, Dr. J. Da, on quinoidine in intermittent fever 295
Corson, Dr. Hiram, on epidemic jaundice 387
Creatine in oxaluria, by Dr. G. W. Miltenberger 325
Creatine in the urine, by J. Cheston Morris, M.D. 532
Cubebs in infantile enuresis, Dr. Deiters on 50

D

Davis, Dr. N. S., History of the American Medical Association, notice 551
Death of Dr. Golding Bird 46
" Prof. Edward Forbes 46
" Dr. M. B. Smith 497
" Dr. M. Stillé 556
" Dr. R. L. Bohannan 556
" Dr. E. Bartlett 556
" M. Valleix 556
" G. L. Upshur, M.D. 635
" Professor Johnston 750
Delvaux, Dr. Prosper, on anthelmintic action of quinine 510
Deiters, Dr., on cubebs in infantile enuresis 50
Dickson, Dr. S. H., Elements of Medicine, notice 697
Dictionary of Terms used in Medicine, &c., by Dr. R. D. Hoblyn, notice of 749
Diabetes mellitus, treatment of 251
Dislocation of the Femur treated by manipulation 319
Dock, Dr. Geo., case of fracture of skull 136
Dock, Dr. Geo., on wound of the throat 476
Dock, Dr. Geo., on operations for cataract 583
Drake, Dr. Daniel, on Principal Diseases of Interior Valley of North America, notice 24
Dropsy after scarlatina, by O. C. Gibbs, M.D. 68
Duncan, Dr. M., on pelvic articulations in labor 52

E

Editorial 38, 232, 314, 371, 440
Eisenmann, Dr., on pathology of pancreas 561
Electro-chemistry in therapeutics 238
Elements of Medicine, by S. H. Dickson, M. D., notice of 697
Elkoplasty, extracts from paper on 446
Empyema, case of, by Dr. J. T. Metcalfe 566
Epilepsy cured by a surgical operation 501
Erysipelas and pyæmia 638
Eye, Treatise on Diseases of the, by William Mackenzie, M.D., notice of 702

F

Fermentation and putrefaction, investigation of facts and theories of, by H. Pemberton 251
Foreign Bodies in the Air Passages, Treatise on, by J. D. Gross, M.D., notice of 155
Forget, M., on calculous phthisis 101
Formulary, Pocket, &c., by T. S. Powell, M.D., notice of 493
Fracture of the cranium, by H. P. C. Wilson, M.D. 527

G

Gastrotomy for the removal of an ovarian tumor, by H. H. Smith, M.D. 1
Gastrotomy for the removal of a leaden bar, by Dr. T. B. Neall, 193
Galvanism, removal of metals by 507
Gestation, prolonged 51
Glagow Medico-Chirurgical Society 53
Glycogenia—the secretion of sugar in the human body, by Bernard Henry, M.D. 713
Gout, illustrations of points in history of 251
Green, Dr. Horace, on Injections into Bronchial tubes, notice 204
Green, Dr. Horace, letter to 374

Grimes, J. Stanley, on Phreno-Geology, notice 29
Gries, Dr. Wm., on the ammonio-tartrate of iron 522
Gross, Dr. J. D., Treatise on Foreign Bodies in Air Passages, notice 155
Gross, Dr. J. D., on Diseases of Urinary Bladder, notice 491

H

Hall, Dr. T. B., on removal of portion of lung 75
Hartshorne, Dr. H., on Animal decomposition 451
Hartshorne, Dr. H., on nature and treatment of cholera 321
Hartshorne, Dr. E., case of poisoning with arsenic 707
Hassall, Arthur H., on Microscopic Anatomy, notice of 36
Haskins, Dr. E. B., on Urinary calculi, notice of 429
Hayward, Dr. Geo., Surgical Reports, notice of 416
Heart, Wounds of the, by S. Purple, M.D., notice of 429
Henry, Bernard, M. D., on glycogenia—the secretion of sugar in the human economy 713
Hernia, case of ventral 635
Hoblyn, Dr. R. D , Dictionary of Terms used in Medicine, &c., notice 749
Holmes, Dr. O. W., on puerperal fever, notice of 162
Hooker, Dr. Worthington, Human Physiology, notice of 36
Horner, Dr. G. R. B., on Diseases of Seamen, notice of 37
Hooping cough, by Dr. L. Turnbull, 461
Huff, Dr. G., on removal of metals from the system by galvanism 507
Hullihen's ear syringe, notice of 44
Human Physiology, by Worthington Hooker, M. D., notice of 36
Hydrophobia, two cases in Smyrna 504

I

Imperforate rectum, case of, by R. A. F. Penrose, M.D., 586
Infant Therapeutics, Dr. John B. Beck's Treatise on, notice of 232
Injections into Bronchial Tubes, &c., by Horace Green, M. D., notice of 204
Intestinal stricture, removal of, by Dr. Van Drommelin, 183
Intestinal perforation in typhoid fever, curability of 235
Iodide of starch, digestibility of, by Dr. Jütte 589
Iron, use of the ammonio-tartrate of, by Wm. Gries, M.D., 522

J

Jameson, Dr. H. G., Treatise on Cholera, notice 549
Jaundice, epidemic, in Montgomery county, by Dr. Corson 387
Jewell, Dr. Wilson, on Mortality of Philadelphia, for—
Oct., Nov., and Dec., 1854 78
Jan., Feb. and March, 1855 302
April, May and June, 1855 479
July, Aug. and Sept., 1855 688
Jobert, M., on cold abscesses 562
Jones, C. Handfield, Manual of Pathological Anatomy, notice 117
Jütte, Dr., on digestibility of iodide of starch 589

K

Kirkbride, Dr. Thos. S., on Construction of Hospitals, notice 314
Kirkpatrick, Prof. J. A., Meteorological observations, 64, 127, 128, 192, 256, 320, 386, 450, 514, 578, 642, 706, 762
Kirkpatrick, Prof. Jas. A., on meteorology 534

L

Labor, induction of, at the seventh month 531
Laurence, Dr. John Zachariah, on the Diagnosis of Surgical Cancer, notice 745
Lee, Dr. Henry, on old stricture of the urethra 59
Leucorrhœa, Dr. Tyler Smith on Pathology of, &c., notice 425
Lewis, Dr. F. W., cases of albuminuria 7
Lexicon, Medical, by D. Meredith Reese, M.D., notice of 493
Lightning, enquiry into number of victims by M. Boudin 94
Littell, Dr., note on secondary variolous ophthalmia 129
Lupulin as an anaphrodisiac 250

M

Mackenzie, Dr. Wm., Treatise on Diseases of the Eye, notice 702
Malformation of the colon and head, case of, by Dr. W. Walters 724
Manual of Clinical Medicine, by T. H. Tanner, M.D., notice of 705

McSherry, Dr. Richard, on amputation of the thigh 316
Meat juice, preserved, by Dr. Christison 189
Medical Jurisprudence, a Treatise on, by Francis Wharton, and Moreton Stillé, M. D., notice of 728
Meigs, Dr. J. F., case of fatal cholera morbus 402
Meigs, C. D., on the Nature, &c., of Child-bed Fevers, notice of 18
Meningitis, analysis of twenty-one cases of 240
Meteorological Observations—
For November, 1854 64
" the year 1854 128
" December, 1854 127
" January, 1855 192
" February, 1855 256
" March, 1855 320
" April, 1855 386
" May, 1855 450
" June, 1855 514
" July, 1855 578
" August, 1855 642
" September, 1855 706
" October, 1855 762
Meteorology—Mason's hygrometer 534
Metcalfe, Dr. John T., on empyema 566
Microscopic Anatomy of Human Body, by Arthur Hill Hassall, notice of 36
Miltenberger, Dr. G. W., on creatine in oxaluria 325
Mineral springs of the United States, by Dr. John Bell 333, 394
Mineral Springs of the United States, Treatise on, by Dr. John Bell, notice of 492
Morris, Dr. J. C., on contagion in puerperal fever 327
Mortality of Philadelphia—
For Oct., Nov. and Dec., 1854 78
" Jan., Feb. and March, 1855 302
" April, May and June, 1855 479
" July, Aug. and Sept., 1855 688
Mortality, British, in England and the Crimea 750
Montgomery, Dr. W. F., illustrations of the influence of pregnancy in controlling or retarding the development of certain diseases 760
Monograph on Mental Unsoundness, by F. Wharton, notice of 487
Morris, Dr. J. C., on creatine in the urine 532
Murdock, Dr. J. M., case of tracheotomy 182
Musket ball in bladder, by J. W. Robinson, M.D. 328
Myers, W. H., case of opium eating cured 139

N

Nature in Disease, by J. Bigelow, M.D., notice of 27
Natural labor in a girl 13 years of age, by Dr. W. S. Stoakeley 203
Nævi, cutaneous, cured by iodine paint 506
Neal, Dr. T. B., case of gastrotomy 193
Neill, Dr. John, on a new form of cataract needle 332
Neill, Dr. John, on a new apparatus for fractures of leg 579
Neill, Dr. John, case of polypi of the conjunctiva 196
New York Academy of Medicine, proceedings of 494
Northern Medical Association, election of officers, &c. 127

O

Obesity in a child 408
Obstetrical supporter, notice of 233
Obstetrical Medicine, Principles and Practice of, by Dr. Ramsbotham, notice of 346
Opium eating cured, case of, by W. H. Myers 139
Ovariotomy, synopsis of thirty cases of 317
Oxyde of carbon as a poison, by M. Adrian Chenol 186

P

Pancreas, Dr. Eisenmann on pathology of 561
Paralysis, Clinical Lectures on, by R. B. Todd, M.D., notice 551
Paris, medical news in, by R. A. Kinlock, M.D., 58
Parker, Langston, on Syphilitic Diseases, notice 26
Parker, E. A., M.B., on physical diagnosis in fevers 47
Pathological Anatomy, Manual of, by C. H. Jones, notice of 117
Pathological Anatomy, Outlines of, by Dr. Charles Wedl, notice of 147
Pathological Anatomy, Manual of, by C. Rokitansky, notice of 631
Pemberton, H., on fermentation and putrefaction 257
Peritonitis in typhoid fever, without perforation 237
Penrose, Dr., on cephalic presentation 405

Penrose, Dr., case of imperforate rectum 586
Pennsylvania State Medical Society 751
Percyanide of mercury in syphilitic ulceration of tongue 641
Phagedæna, treatment of hospital 247
Philadelphia County Medical Society, election of officers 126
Phosphate of lime, inefficacy of 590
Physical Diagnosis in Fevers, by E. A. Parker, M.B. 47
Phreno-Geology, by J. Stanley Grimes, notice of 29
Physician's Visiting List, notice of 705
Plague, origin and disappearance, by Aug. T. Stamm 515
Powell, Dr. T. S., Pocket Formulary, &c., notice of 493
Poisoning by nitrate of potash 244
Poisoning by cantharides 249
Polypi of the conjunctiva, case of, by Dr. John Neill 196
Potash, treatment of acute rheumatism by bicarbonate of 253
Post-mortem appearances of an old lady 50
Principles of Physiology, for use of Schools, by Drs. Comstock and Comings, notice of 171
Principles of Human Physiology, with their chief applications to psychology, pathology, therapeutics, hygiene and forensic medicine, by Wm. B. Carpenter, M.D., notice of 744
Practice of Medicine, Dr. Wood's Treatise on, notice of 227
Prescriptions, Book of, by Henry Beasley, M.D., 747
Pregnancy, illustrations of the influence of, in controlling or retarding the development of certain diseases, by W. F. Montgomery, M.D. 760
Proceedings of the American Medical Association 348
Proceedings of Medical Society of State of Pennsylvania 431
Puerperal fever as a private pestilence, by O. W. Holmes, notice of 168
Purple, Dr. Samuel, Observations on Wounds of the Heart, notice 429

Q

Quinoidine in intermittent fever, by J. DaCosta 295

R

Ramsbotham's Principles and Practice of Obstetrics, notice of 346
Rand, Dr. B. Howard, Outline of Medical Chemistry, notice 345
Reese, Dr. D. M., Medical Lexicon, notice 493
Removal of portion of left lung, by T. B. Hale, M.D. 75
Resection of the right elbow, by H. H. Smith, M.D. 65
Resection of the tarsal and metatarsal bones, by H. H. Smith, M.D. 131
Resuscitation of a still-born child 640
Ricinus communis as an emmenagogue, &c. 246
Rheumatism, treatment of acute, by the bicarbonate of potash 253
Rheumatism, contractions from 636
Robinson, Dr. Jas. W., case of musket ball in bladder 328
Rokitansky, Carl, Manual of Pathological Anatomy, notice 631
Ruschenberger, Dr. W. S. W., on medical topography, climate and yellow fever of Rio Janeiro 643

S

Salæratus, medicinal effects of 141
Salutatory—editorial 38
Sanitary Commission on Yellow Fever, Report of, by Dr. E. H. Barton, &c., notice of 309
Sanitarium in East Florida 552
Sausage poison, nature of 101
Scarlatina, Dr. Tweedy's observations on 557
Schelling, Dr., report on vaccination in the Prussian army 409
Schreier, Dr. F., on the use of the tinct. ferr. chlorid. in uterine hemorrhages 725
Secret remedies 567
Sickness and Mortality on board of Emigrant Ships, Report on, notice of 169
Simon, John, F.R.S., on Sanitary Condition of London, notice of 342
Skey, Mr. Frederick C., on ulceration of the legs 175
Skull, fracture of, by Dr. Geo. Dock 136
" " by Prof. J. Syme 174
Smith, Sidney, as a country doctor 562
Smith, Dr. H. H., on gastrotomy for the removal of an ovarian tumor 1
Smith, Dr. H. H., on resection of the right elbow 65
Smith, Dr. H. H., on resection of the tarsal and metatarsal bones 131
Smith, Dr. H. H., appointment of 385

Smith, Tyler, on Pathology of Leucorrhœa, notice of 425
Stamm, Theod. Aug., on the plague 515
Statistics, notes on the application of, to questions in medical science, by W. P. Alison, M.D. 752
Stick or swab-handle in stomach, Garcia's case of 91
Stillé, Dr. Moreton, appointment as lecturer 173
Stillé, Dr. Moreton, death of 555
Stoakley, Dr. W. S., case of labor in a girl 13 years of age 203
Surgical Reports, &c., by Dr. Geo. Hayward, notice of 416
Syme, Prof. James, on fracture of the skull 174
Syphilitic Diseases, Treatment of, by Langston Parker, Surgeon, notice of 26

T

Therapeutics, notices of hospital 569
Todd, Dr. R. B., Clinical Lectures on Paralysis, notice 551
Toe-nail, on the ingrowing 512
Topical Medication of the Larynx, by Eben Watson, M.D., notice of 204
Transactions of New York State Medical Society 632
Transactions of the American Medical Association, notice of 37,108,749
Tracheotomy, case of, by J. M. Murdock, M.D. 182
Tumbler taken from rectum 563
Turnbull, account of Dr. Alex. 377
Turning, two cases of, not accomplished 244
Turnbull, Dr. L., on hooping cough 461
Tweedy, Dr. H., on scarlatina 557

U

Ulceration of the Os Uteri, enquiry into importance of, by Dr. C. West, notice of 166
Ulceration of the legs, lecture on, by Frederick C. Skey, Esq. 175
Upshur, death of Dr. Geo. L. 635
Urinary Calculi, Analysis of, by Dr. E. B. Haskins, notice of 429
Urinary Bladder, Treatise on Diseases of, by S. D. Gross, M.D., notice of 491
Uterus, exfoliation of mucous membrane of, by Tyler Smith, M.D. 499
Uterine hemorrhages, use of the tinct. ferr. chlorid. in, by Dr. F. Schreier 725

V

Vaccination, report on, in the Royal Prussian Army, by Dr. Schelling 409
Varicose ulcers, use of the pad in, by R. Barnwell 504
Virchow, Rudolph, on calcareous metastases 5€4

W

Watson, Dr. Eben, on Topical Medication of Larynx, notice 204
Walters, Dr. W., case of malformation of the colon and heart 724
Wedl, Dr. Charles, Outlines of Pathological Anatomy, notice 147
West, Dr. C., Enquiry into importance of Ulceration of the Os Uteri, notice 166
Wharton, Francis, on Mental Unsoundness, notice 487
What to observe at the Bedside, &c., Treatise on, notice of 171
Wilson, Dr. H. P. C., on fracture of cranium 527
Women, a Practical Treatise on Diseases of, by S. Ashwell, notice of 419
Wood, Prof. Geo. B., Treatise on the Practice of Medicine, notice 227
Wound of the throat, by Geo. Dock, M.D. 476

Y

Yellow fever, progress of, in Virginia 553
Yellow fever, Treatise on, by R. La Roche, M.D., notice of 590
Yellow fever, cause and prevention of, by Dr. E. H. Barton 683
Yellow fever in Norfolk and Portsmouth 633
Yellow fever of Rio de Janoiro, by W. S. W. Ruschenberger, M.D. 643

